# Supplementary material for: Unraveling Racial Disparities in Papillary Thyroid Cancer: A Comparative Bulk RNA-Sequencing Gene Expression Analysis
Source: Curr Oncol. 2025 May 29;32(6):315. doi: 10.3390/curroncol32060315 (PMC12191872; doi:10.3390/curroncol32060315)
Supplement: Supplementary file 1 [file curroncol-32-00315-s001.zip › Supplemental Figure Legends.pdf]

Supplemental Figure Legends

Figure S1*:* Gene expression levels of *ATP6V0E2* (A), *CD38* (B), *EAF2* (C), *GTF2H3* (D), *HYOU1* (E), and *KCNQ1* (F) in thyroid carcinoma (THCA) samples from The Cancer Genome Atlas (TCGA), comparing Caucasian, African American, and Asian patients. All six genes show significantly decreased expression in THCA compared to control tissue across racial groups ($p < 0.05$ (*); $p < 0.01$ (**); $p < 0.0001$ (***).

Figure S2*:* Gene expression levels of *LAX1* (A), *MIR20SHG* (B), *PDK1* (C), *PGF* (D), *PIWIL1* (E), and *PPFIA2* (F) in thyroid carcinoma (THCA) samples from The Cancer Genome Atlas (TCGA), comparing Caucasian, African American, and Asian patients. *LAX1, PDK1, PGF*, and *PPFIA*2 show significantly decreased expression in THCA compared to control tissues across racial groups, while *MIR20SHG* and *PIWIL1* show significantly increased expression in THCA across racial groups ($p < 0.05$ (*); $p < 0.01$ (**); $p < 0.0001$ (***).

Figure S3. Gene expression levels of *HHEX* (A), *RICTOR* (B), *RPL17* (C), *SLC16A9* (D), *SOD3* (E), and *TXNDC5* (F) in thyroid carcinoma (THCA) samples from The Cancer Genome Atlas (TCGA), comparing Caucasian, African American, and Asian patients. *HHEX, RICTOR, RPL17, SOD3,* and *TXNDC5* show significantly decreased expression in THCA compared to control tissues across racial groups. For *SLC16A9*, there was significantly increased expression in African American THCA and significantly decreased expression in Caucasian and Asian THCA compared to control tissue ($p < 0.05$ (*); $p < 0.01$ (**); $p < 0.0001$ (***).

Figure S4*:* Gene expression levels of *XBP1* (A), *ZBED2* (B), and *ZNF700* (C) in thyroid carcinoma (THCA) samples from The Cancer Genome Atlas (TCGA), comparing Caucasian, African American, and Asian patients. Effect of gene expression levels of *HYOU1* (D), *PDK1* (E), and *PPFIA2* (F) on THCA patient survival. *XBP1, ZBED2*, and *ZNF700* show significantly decreased expression in THCA compared to control tissues across racial groups. THCA patients with High expression of *HYOU1, PDK1,* and *PPFIA2* show significantly decreased survival probability compared to THCA patients with Low Medium expression ($p < 0.05$ (*); $p < 0.01$ (**); $p < 0.0001$ (***).
