# Supplementary material for: Unraveling Racial Disparities in Papillary Thyroid Cancer: A Comparative Bulk RNA-Sequencing Gene Expression Analysis
Source: Curr Oncol. 2025 May 29;32(6):315. doi: 10.3390/curroncol32060315 (PMC12191872; doi:10.3390/curroncol32060315)
Supplement: Supplementary file 1 [file curroncol-32-00315-s001.zip › Table S1.pdf]

Table S1: Patient Demographic with papillary thyroid cancer.

Race	Age	Sex	Staging	PTC Subtype
White/Caucasian	82	M	pT1bpNX	Classical
White/Caucasian	68	F	pT1apN1b	Classical
White/Caucasian	76	F	pT2pN1b	Classical
White/Caucasian	55	F	pT1bpN1a	Classical
White/Caucasian	51	F	pT1bpN1b	Classical
Asian Indian	56	F	pT1bpN1a	Classical
Asian Indian	42	F	pT1apN0a	Classical
Asian	76	M	pT1bpNX	Classical
Asian	57	M	pT2pNX	Classical
Asian Filipino	49	F	pT1bpN0a	Classical
Hispanic	58	F	pT4bpN1a	Classical
Hispanic	27	M	pT1bpN1b	Classical
Hispanic	68	F	pT1apN1a	Classical
Hispanic	33	F	pT1bpNX	Classical
Hispanic	43	F	pT1bpN0a	Classical
African American	69	M	pT3bpN1b	Classical
African American	57	F	pT2pNX	Classical
African American	46	M	pT1apN0a	Classical
African American	64	F	pT1bpN0a	Classical
African American	21	F	pT3apNX	Classical
