# Supplementary material for: Unraveling Racial Disparities in Papillary Thyroid Cancer: A Comparative Bulk RNA-Sequencing Gene Expression Analysis
Source: Curr Oncol. 2025 May 29;32(6):315. doi: 10.3390/curroncol32060315 (PMC12191872; doi:10.3390/curroncol32060315)
Supplement: Supplementary file 1 [file curroncol-32-00315-s001.zip › Table S2.pdf]

Table S2. Differential expressions of top 10 upregulated and downregulated genes

Gene Name	Fold Change	EA Mean	Asian Mean	*P* value
ENSG00000285628	6.60985	16.03072	0	0.02547
IGHV5-10-1	5.04973	8.22352	0.22814	0.04303
ENSG00000284337	5.02373	12.23614	0.34957	0.00118
ENSG00000272647	4.76155	4.57611	0	0.02737
SAA1	4.27176	16.10916	0.8116	0.00104
SYNGAP1-AS1	4.17271	3.02379	0	0.04575
IGKV5-2	4.11503	6.62894	0.42266	0.00639
PLA2G2A	3.88825	135.25294	9.14611	3.00E-05
ENSG00000262730	3.64794	2.16507	0	0.02407
IGHV4-4	3.52643	34.67205	2.9943	0.0359
ENSG00000244380	3.4487	1.58046	0	0.01768
ENSG00000258566	3.34947	1.74476	0	0.03378
RPS7P14	3.34192	2.59779	0.22814	0.04033
IGHV2-70	3.28017	29.52315	3.11299	0.02361
MCEMP1	3.16475	52.68566	5.9385	2.00E-05
ENSG00000256533	3.15856	2.41783	0.14988	0.0237
PMCH	3.02073	1.94811	0.12793	0.03222
GNL3LP1	2.9339	13.36224	1.7972	0.0016
ENSG00000259001	2.92037	3.68003	0.42768	0.03477
ENSG00000289381	2.91979	5.98267	0.8116	0.01069
BMS1P7	-7.49329	0	34.46564	0.00904
USP17L3	-7.24833	0	29.0263	8.00E-05
RPL3P4	-7.03542	0.53464	84.35654	3.00E-05
ADH5P5	-5.5209	0.35143	13.90844	0.01385
ENSG00000288656	-5.43263	0	8.24988	0.00056
IGKV3D-15	-4.88142	0	5.63939	0.04064
ENSG00000279905	-4.61453	0	4.66009	7.00E-05
TM4SF19-DYNLT2B	-4.56392	0	4.52904	0.00429
ENSG00000258472	-4.36005	0	3.90424	0.00619
KRT5	-4.24782	26.34571	499.88974	2.00E-05
CNMD	-4.24605	20.50015	394.26811	0.00022
OR7E84P	-4.10442	0.18604	5.12685	0.01226
ENSG00000286319	-4.07202	0	3.19655	0.00318
PSPHP1	-4.06045	0	3.07306	0.00019
ENSG00000226432	-4.05158	1.36102	23.69471	0.00122
MTCO1P12	-3.9099	1.98971	32.00478	0.00236
ENSG00000285476	-3.88022	0.08128	4.09457	0.02931
SNAI3-AS1	-3.87108	0	2.88553	0.0059
IGHV1-69-2	-3.79924	0.63402	6.50387	0.02203
MT1G	-3.77516	17.98598	249.39477	0.00013
