# Supplementary material for: Unraveling Racial Disparities in Papillary Thyroid Cancer: A Comparative Bulk RNA-Sequencing Gene Expression Analysis
Source: Curr Oncol. 2025 May 29;32(6):315. doi: 10.3390/curroncol32060315 (PMC12191872; doi:10.3390/curroncol32060315)
Supplement: Supplementary file 1 [file curroncol-32-00315-s001.zip › Table S3.pdf]

Table S3. Differential expressions of top 10 downregulated and upregulated genes

Gene Name	EA-African Fold Change	EA Mean	African Mean	p value
TSPY4	-18.07122	0	5.37287	1.00E-05
IGKV3D-15	-8.35433	0	62.62879	0.00043
BMS1P7	-6.97132	0	23.99177	0.01519
IGHV3OR16-10	-6.5528	0.40642	50.95966	0.02077
IGHV3-64D	-6.48695	3.13758	286.48649	0.00146
IGKV1D-13	-6.42522	0.82543	76.77103	1.00E-05
IGHV3-33	-6.03885	168.664	11083.33671	1.00E-05
IGKV1D-39	-5.72724	134.42743	7117.09769	1.00E-05
IGHV3-41	-5.63536	0	9.57341	0.00034
IGHV3-74	-5.57498	94.59098	4504.98397	4.00E-05
IGHV3-73	-5.47315	6.6362	290.5121	0.00015
IGKV3-7	-5.40204	1.28257	51.84574	0.00041
IGHV4-39	-5.40038	54.85083	2310.28202	7.00E-05
USP17L20	-5.39069	2.92623	126.93603	0.0297
IGKV1-39	-5.2866	2.59629	99.00536	0.01156
IGHV4-4	-5.15535	34.67205	1230.56089	0.0017
IGLV7-46	-5.14447	1.53387	50.07323	0.0014
IGLC1	-5.08619	344.75652	11705.55047	1.00E-05
IGKV3-15	-5.08519	56.96046	1933.56545	1.00E-05
IGHV2-26	-5.06544	4.57226	147.62014	1.00E-05
ENSG00000167774	7.14768	25.12735	0	0.01236
ENSG00000283537	7.05368	23.64929	0	0.00017
ENSG00000267149	5.77443	9.68151	0	0.01751
DEFA1B	5.50459	8.0088	0	0.03037
ENSG00000173366	4.8767	5.31117	0	0.00106
GUSBP15	4.43155	8.98117	0.42885	0.02579
ENSG00000259164	4.40651	3.77085	0	0.02689
ENSG00000284337	4.20405	12.23614	0.58139	0.00454
ENSG00000262880	4.09805	3.13526	0	0.01356
PHOX2B-AS1	4.07745	2.98977	0	0.02723
ENSG00000226160	3.86469	2.58727	0	0.00662
CXorf49	3.84063	11.82525	0.8577	0.0127
FAM237B	3.6461	2.22646	0	0.01443
TRBV7-7	3.58029	3.16548	0.15115	0.00639
ENSG00000251246	3.52822	3.0784	0.15115	0.04628
ENSG00000272601	3.48748	7.56434	0.60461	0.0093
DNAJC19P9	3.3806	8.78997	0.75577	0.00508
LINC00467	3.33263	1.79951	0	0.03553
YWHAQP6	3.28126	2.63052	0.15115	0.01996
ARHGAP44-AS1	3.26803	1.86972	0	0.03582
