# Supplementary material for: Unraveling Racial Disparities in Papillary Thyroid Cancer: A Comparative Bulk RNA-Sequencing Gene Expression Analysis
Source: Curr Oncol. 2025 May 29;32(6):315. doi: 10.3390/curroncol32060315 (PMC12191872; doi:10.3390/curroncol32060315)
Supplement: Supplementary file 1 [file curroncol-32-00315-s001.zip › Table S4.pdf]

Table S4: Differential expressions of top 10 upregulated and downregulated genes

Gene Name	Fold Change	EA Mean	Hispanic Mean	*p* value
ENSG00000144785	7.04573	26.13857	0	0.01139
ENSG00000167774	6.98665	25.12735	0	0.02185
ENSG00000268400	6.48278	17.7134	0	0.01906
ENSG00000264324	5.60423	9.65893	0	0.01906
GUSBP15	5.48949	8.98117	0	0.0105
ENSG00000256407	5.04837	6.62157	0	0.0234
ENSG00000260537	5.03267	6.26551	0	0.00722
ENSG00000267633	4.93255	5.98539	0	0.01129
NPIPA3	4.92889	102.66242	3.37203	2.00E-05
ENSG00000259164	4.24549	3.77085	0	0.04651
ABCB10P4	4.24011	3.84661	0	0.03792
ENSG00000251246	3.94427	3.0784	0	0.03793
ENSG00000274642	3.83122	4.4093	0.13663	0.03239
ENSG00000270401	3.77962	2.77951	0	0.02816
ENSG00000284337	3.76696	12.23614	0.93914	0.01564
ENSG00000228327	3.75023	2.65823	0	0.04966
SEMA6A-AS1	3.59405	2.40461	0	0.02823
ENSG00000230387	3.4476	2.34467	0	0.0092
ENSG00000287237	3.38548	2.03548	0	0.01665
PMCH	3.33458	1.94811	0	0.02836
IGKV3D-15	-6.91922	0	23.17167	0.00562
TM4SF19-DYNLT2B	-5.65925	0	9.77289	0.00062
USP17L20	-5.54353	2.92623	141.13572	0.03479
IGHV7-4-1	-4.96693	2.33793	70.6393	0.00042
PSPHP1	-4.96571	0	5.68447	1.00E-05
SFTPA1	-4.7488	8.29716	222.51839	6.00E-04
ENSG00000286319	-4.6241	0	4.59541	0.00118
ENSG00000260122	-4.51932	0	4.45028	0.02883
IGHV3-64D	-4.3269	3.13758	64.11854	0.04507
ELF2P4	-4.25077	0.24385	8.98407	0.00099
RFPL4B	-4.14495	0.35143	5.26307	0.0053
IL9RP3	-4.11249	0.66844	7.66791	0.00143
ENSG00000283427	-4.07483	0.95103	11.00368	2.00E-04
USP17L3	-3.98252	0	3.03017	0.0428
ENSG00000276888	-3.9613	0	3.0478	0.04294
SNAI3-AS1	-3.82905	0	2.67374	0.00954
ENSG00000279905	-3.81492	0	2.52344	0.00214
ENSG00000266677	-3.81291	0.69329	9.09124	0.00077
RPL3P4	-3.80433	0.53464	8.95875	0.03374
ENSG00000274835	-3.79341	0.08128	3.93709	0.00348
