# Supplementary material for: Unraveling Racial Disparities in Papillary Thyroid Cancer: A Comparative Bulk RNA-Sequencing Gene Expression Analysis
Source: Curr Oncol. 2025 May 29;32(6):315. doi: 10.3390/curroncol32060315 (PMC12191872; doi:10.3390/curroncol32060315)
Supplement: Supplementary file 1 [file curroncol-32-00315-s001.zip › Table S5.pdf]

Table S5: Differential expressions of top 10 upregulated and downregulated genes

Gene Name	Asian African Fold Change	Asian Mean	African Mean	*p* value
ENSG00000283537	6.64718	17.69577	0	4.00E-04
RPL3P4	5.85138	84.35654	1.37217	0.00023
ENSG00000167774	5.77426	9.66185	0	0.04353
DEFA1B	5.43314	7.64022	0	0.03245
DMRTC1	5.25843	6.767	0	0.04378
MTCO1P12	5.08845	32.00478	0.9439	0.00019
ENSG00000267149	5.05784	5.89346	0	0.03768
CNMD	5.01806	394.26811	12.1247	1.00E-05
ENSG00000237188	4.9449	5.46312	0	0.02597
TM4SF19-DYNLT2B	4.67707	4.52904	0	0.00343
ENSG00000226160	4.51217	4.0601	0	0.0012
KRT5	4.36036	499.88974	24.41292	1.00E-05
ENSG00000259164	3.96345	2.80157	0	0.04681
USP17L18	3.93339	29.14558	1.81615	0.00128
USP17L3	3.82009	29.0263	2.06808	0.02089
ENSG00000255026	3.79901	3.71457	0.15115	0.02142
ENSG00000284048	3.71741	2.34119	0	0.01949
LINC00467	3.59252	2.18496	0	0.02122
OR4M2	3.48445	11.37497	0.94055	0.00158
ENSG00000236299	3.39748	15.82233	1.49104	0.00372
IGHV3-64D	-9.10019	0.52789	286.48649	3.00E-05
IGKV2D-40	-8.48282	0	58.96629	0.00935
IGHV5-10-1	-7.98532	0.22814	62.30549	0.00131
IGLV4-60	-6.65686	0.22814	24.87308	2.00E-05
ENSG00000285628	-6.6121	0	16.09228	0.02539
IGHV4-61	-6.57347	17.89687	1699.3237	2.00E-05
IGHV3OR16-10	-6.41201	0.58347	50.95966	0.02314
IGLV4-69	-6.33224	0.70046	56.51951	1.00E-05
IGHV1-58	-6.13547	1.18948	85.58576	0.00207
IGKV3-7	-6.1071	0.77872	51.84574	1.00E-04
IGHV2-70	-5.86994	3.11299	178.92965	5.00E-05
IGLV1-51	-5.78199	19.52853	1077.17093	2.00E-05
IGHV3-69-1	-5.62414	6.83777	336.81058	0.00041
IGLV1-44	-5.53688	27.92408	1294.75481	1.00E-05
IGLV9-49	-5.53363	0.79553	34.89153	2.00E-05
IGHV3-73	-5.40077	6.79002	290.5121	0.00017
HSFX2	-5.19528	1.16176	43.11682	1.00E-04
IGLV8-61	-5.17989	1.0617	38.29944	5.00E-05
IGHV3-49	-5.16041	5.05513	180.70306	1.00E-05
IGHV2-26	-5.15366	4.15432	147.62014	1.00E-05
