# Supplementary material for: Unraveling Racial Disparities in Papillary Thyroid Cancer: A Comparative Bulk RNA-Sequencing Gene Expression Analysis
Source: Curr Oncol. 2025 May 29;32(6):315. doi: 10.3390/curroncol32060315 (PMC12191872; doi:10.3390/curroncol32060315)
Supplement: Supplementary file 1 [file curroncol-32-00315-s001.zip › Table S6.pdf]

Table S6: Differential expressions of top 10 upregulated and downregulated genes

Gene Name	Asian-Hispanic Fold Change	Asian Mean	Hispanic Mean	*p* value
ENSG00000260537	7.08277	26.86475	0	0.00014
MTCO1P12	5.76941	32.00478	0.62699	0.00025
ENSG00000144785	5.59727	9.58507	0	0.0447
ENSG00000288656	5.38476	8.24988	0	0.00154
ENSG00000264324	5.22665	7.36229	0	0.02881
ENSG00000286273	4.86441	5.76445	0	0.00458
ST7-AS1	4.8064	5.64605	0	8.00E-05
ENSG00000276418	4.71615	5.23738	0	0.03865
ENSG00000234353	4.70895	5.20481	0	0.01859
OR7E84P	4.69228	5.12685	0	0.00854
ABCB10P4	4.52561	4.57505	0	0.02592
ENSG00000285476	4.36246	4.09457	0	0.02358
PDXP-DT	4.3499	3.97456	0	0.00057
ENSG00000270401	4.31462	3.99122	0	0.01112
ENSG00000235738	4.28683	3.88436	0	0.01219
RPS9P1	4.21966	3.69103	0	0.01398
ENSG00000255026	4.21506	3.71457	0	0.01772
ACTG1P15	4.1364	3.52655	0	0.03438
ENSG00000289353	4.04933	3.26967	0	0.00322
PTGES3L	4.01611	5.43087	0.31305	0.00388
IGHV3-64D	-6.94014	0.52789	64.11854	0.00238
ENSG00000285628	-6.82037	0	18.62975	0.02887
ENSG00000277998	-6.11491	0	11.42355	0.04903
MRPS36P1	-6.04916	0	10.95583	0.00213
ENSG00000285816	-5.25809	0	6.29901	0.02049
ANKRD26P1	-5.00474	0	5.06147	0.00035
IGLV4-60	-4.88356	0.22814	7.03407	0.00265
IGHV2-70	-4.64535	3.11299	76.35206	0.00238
HSFX2	-4.6061	1.16176	28.55896	0.00106
KIAA1671-AS1	-4.38673	0	3.27915	0.02857
IGHV4-4	-4.37624	2.9943	62.23346	0.01377
ENSG00000267110	-4.33291	0	3.24818	0.01533
IGKV1-9	-4.16464	7.34371	131.98811	0.00511
IGLV2-23	-3.90614	3.51905	52.94717	0.00709
GXYLT1P4	-3.87768	0.14988	3.67659	0.04618
IGKV3-11	-3.77351	14.38996	196.26658	0.00232
IGHV3-48	-3.6912	22.05238	284.83627	0.00161
ENSG00000257390	-3.64076	1.17281	14.9474	0.00464
IGLV3-27	-3.63447	1.20138	14.63297	0.03819
IGHV3-49	-3.61128	5.05513	61.77084	0.00305
