# Supplementary material for: Unraveling Racial Disparities in Papillary Thyroid Cancer: A Comparative Bulk RNA-Sequencing Gene Expression Analysis
Source: Curr Oncol. 2025 May 29;32(6):315. doi: 10.3390/curroncol32060315 (PMC12191872; doi:10.3390/curroncol32060315)
Supplement: Supplementary file 1 [file curroncol-32-00315-s001.zip › Table S7.pdf]

Table S7. Differential expressions of top 10 upregulated and downregulated genes

Gene Name	African-Hispanic Fold Change	African Mean	Hispanic Mean	*p* value
IGHV3OR16-10	8.00919	50.95966	0	0.00861
IGLV1-47	5.87123	1856.75848	31.78414	2.00E-05
ENSG00000260537	5.33417	8.06131	0	0.00428
IGLV1-36	5.25632	12.84217	0.31305	0.00064
IGHV4-59	5.08509	7768.07325	228.83234	2.00E-05
IGHV3-74	5.03178	4504.98397	137.73093	0.00044
IGKV4-1	4.92053	804.24373	26.61774	3.00E-05
IGKV1-17	4.82468	979.67388	34.62363	0.00118
IGHV1-46	4.81631	1056.86804	37.53692	0.00015
IGLV10-54	4.78229	29.33801	1.07757	0.00033
NPIPA3	4.73449	89.58701	3.37203	4.00E-05
IGHV3-33	4.72827	11083.33671	418.0784	0.00108
SEPTIN7P3	4.68653	21.68202	0.79854	0.00345
IGLV1-51	4.6519	1077.17093	42.8853	0.00125
IGHV1-69D	4.63283	1659.09682	66.8422	0.00216
IGHV4-34	4.6172	1502.41603	61.27348	0.00013
IGHV4-61	4.60806	1699.3237	69.66734	0.00461
ABCB10P4	4.59262	4.77156	0	0.02386
IGKV1D-13	4.58618	76.77103	3.24726	0.00132
IGHV4-28	4.57672	286.68315	12.07603	0.00014
ENSG00000283537	-7.64483	0	35.45026	1.00E-04
DMRTC1	-7.15694	0	25.25535	0.00901
ENSG00000173366	-6.08012	0	11.98734	7.00E-05
TM4SF19-DYNLT2B	-5.7724	0	9.77289	0.00048
ENSG00000257432	-4.34096	0	3.58766	0.03833
ENSG00000257390	-4.32736	0.73975	14.9474	0.0016
ENSG00000236299	-4.17689	1.49104	27.15825	0.00066
ENSG00000226160	-4.14595	0	3.05751	0.00478
ENSG00000262880	-3.9105	0	2.67681	0.02495
ENSG00000270008	-3.86835	0	2.52344	0.03116
KRT222	-3.70584	0.42885	5.4027	0.02844
HBD	-3.67728	0.50789	5.14589	0.00229
OR4M2	-3.65497	0.94055	12.98493	0.00152
ENSG00000250762	-3.55427	0	2.12632	0.01409
NUTF2P3	-3.51433	0	2.14088	0.02445
PROK2	-3.45753	0	1.9508	0.01336
ENSG00000230570	-3.38808	0.27908	4.15059	0.01933
ENSG00000284048	-3.37398	0	1.83849	0.04678
ARHGAP44-AS1	-3.36066	0	1.87917	0.03857
ENSG00000288796	-3.16678	0.75577	7.88693	0.00407
